# The Piezo-Hyperthermophilic Archaeon *Thermococcus piezophilus* Regulates Its Energy Efficiency System to Cope With Large Hydrostatic Pressure Variations

**DOI:** 10.3389/fmicb.2021.730231

**Published:** 2021-11-03

**Authors:** Yann Moalic, Jordan Hartunians, Cécile Dalmasso, Damien Courtine, Myriam Georges, Philippe Oger, Zongze Shao, Mohamed Jebbar, Karine Alain

**Affiliations:** ^1^Univ Brest, CNRS, Ifremer, Laboratoire de Microbiologie des Environnements Extrêmes LM2E, UMR 6197, IUEM, Plouzané, France; ^2^IRP 1211 MicrobSea, Sino-French Laboratory of Deep-Sea Microbiology, LM2E, Plouzané, France; ^3^Université de Lyon, INSA Lyon, CNRS UMR 5240, Villeurbanne, France; ^4^Key Laboratory of Marine Biogenetic Resources, The Third Institute of Oceanography SOA, Xiamen, China

**Keywords:** pressure, piezophile, thermococci, hydrothermal, transcriptomics

## Abstract

Deep-sea ecosystems share a common physical parameter, namely high hydrostatic pressure (HHP). Some of the microorganisms isolated at great depths have a high physiological plasticity to face pressure variations. The adaptive strategies by which deep-sea microorganisms cope with HHP variations remain to be elucidated, especially considering the extent of their biotopes on Earth. Herein, we investigated the gene expression patterns of *Thermococcus piezophilus*, a piezohyperthermophilic archaeon isolated from the deepest hydrothermal vent known to date, under sub-optimal, optimal and supra-optimal pressures (0.1, 50, and 90 MPa, respectively). At stressful pressures [sub-optimal (0.1 MPa) and supra-optimal (90 MPa) conditions], no classical stress response was observed. Instead, we observed an unexpected transcriptional modulation of more than a hundred gene clusters, under the putative control of the master transcriptional regulator SurR, some of which are described as being involved in energy metabolism. This suggests a fine-tuning effect of HHP on the SurR regulon. Pressure could act on gene regulation, in addition to modulating their expression.

## Introduction

Seventy-five percent of the ocean volume are subjected to pressures above 10 MPa (Jannasch and Taylor, 1984), described as high hydrostatic pressure (HHP) environments (Oger and Jebbar, 2010). Some “pressure-adapted” microorganisms, called piezophiles, live in these deep marine biosphere ecosystems (Yayanos, 1995). By definition, piezophilic organisms are characterized by a higher growth rate under HHP than at atmospheric pressure. HHP have important impacts onto macromolecules, notably membrane and proteins, and onto physiology and metabolism (Oger and Jebbar, 2010). Piezophilic microorganisms have developed various strategies to cope with HHP. These strategies have mainly been described in psychro-piezophilic bacteria. They include changes in gene expression, switch in metabolism to counteract the loss of biological activity, structural adaptations of macromolecules in order to face HHP and specific stress responses (Simonato et al., 2006; Oger and Jebbar, 2010; Picard and Daniel, 2013; Jebbar et al., 2015).

Although deep oceanic hydrothermal vents are characterized by high temperatures and hydrostatic pressures, very few (hyper)thermophilic piezophilic prokaryotes have been isolated from these ecosystems, and of the available isolates few have been tested for pressure tolerance, mostly due to the inherent technical constraints. Thus, the adaptation mechanisms of (hyper)thermophilic piezophiles remain unwell documented. Compared to psychrophiles, in which pressure and cold adaptation mechanisms may be overlapping, it is easier to independently sift through pressure adaptation mechanisms within (hyper)thermophilic models where high pressures and high temperatures have antagonistic effects (Siliakus et al., 2017). So far, only few (hyper)thermophilic piezophilic or piezosensitive microorganisms have been studied with respect to their response to HHP and most studies have focused on the order *Thermococcales*, a lineage of hyperthermophilic, heterotrophic, sulfur-reducing archaea, some of which are ubiquitous in the hot areas of deep-sea hydrothermal vents. Many studies focused particularly on the piezophiles *Thermococcus barophilus* and *Pyrococ*cus yayanosii (Cario et al., 2015b; Vannier et al., 2015; Michoud and Jebbar, 2016). The transcriptomic response to supra-optimal pressures for growth of *Thermococcus kodakarensis*, a piezosensitive archaeon belonging to *Thermococcales*, resembles a classical stress response. It involves an up-regulation of genes involved in replication, repair and defense mechanisms, and a down-regulation of genes involved in energy production/conversion and in amino acid transport and metabolism. In contrast, the piezophilic taxon *T. barophilus* responds to changes in hydrostatic pressure by some variations in the metabolic pathways used and notably by variations of expression levels of genes coding for transporters, hydrogenases and oxidoreductases (Vannier et al., 2015). At stressful sub-optimal pressures, it also accumulates mannosylglycerate, a compatible solute, probably to help maintain cell volume and homeostasis (Cario et al., 2016). Adaptations at the membrane level (variations of the ratio archaeol/caldarchaeol and of lycopene unsaturations) were also observed in *T. barophilus* to maintain membrane fluidity as a result of pressure changes (Cario et al., 2015a). In addition, proteome adaptations (flexibility linked to reduced hydration water dynamics) of piezophilic species were also reported (Martinez et al., 2016). The obligate piezophile *P. yayanosii* responds to supra- and sub-optimal pressure changes by a major metabolic shift, namely a repression of the H_2_ metabolism and an increase in activity of sulfur-dependent hydrogenases, in addition to changes in the chemotaxis pathway, translation and CRISPR-*cas* gene expressions (Michoud and Jebbar, 2016). Overall, *Thermococcales* respond to pressure variations through structural flexibility and modulation of gene expression.

The hyperthermophilic piezophilic archaeon *Thermococcus piezophilus* strain CDGS^T^ was isolated from the world’s deepest known hydrothermal vent field, at nearly 5,000 m water depth, at the Mid-Cayman rise (Dalmasso et al., 2016b). It has an optimal pressure for growth of 50 MPa and holds the current record of pressure range for growth, growing effectively from atmospheric pressure to at least 120 MPa, and, though with difficulty up to 130 MPa (Dalmasso et al., 2016b). Due to its wide range of growth pressure, it appeared as a good model organism to perform transcriptome-level studies of adaptation to various pressure conditions, as for *T. barophilus* and *P*. yayanosii (Vannier et al., 2015; Michoud and Jebbar, 2016). To address the question of the effects of HHP variations on the transcriptome of *T. piezophilus*, RNA-seq analyses were performed on cells grown at sub-optimal (0.1 MPa), optimal (50 MPa), and supra-optimal (90 MPa) pressures for growth. The selection of these pressures was experimentally determined (growth yields identical to 0.1and 90 MPa and sufficient biomass to perform transcriptomic analysis) (Dalmasso et al., 2016b). Here, by highlighting key gene clusters and the associated metabolic pathways engaged at optimal, sub-optimal and supra-optimal pressures for growth of this hyperthermophilic piezophilic archaeal model, we provide new insights into the molecular responses of piezophiles to pressure variations.

## Materials and Methods

### Medium Culture and Growth Conditions

*Thermococcus piezophilus* strain CDGS^T^ (UBOCC 3296^T^ = ATCC TSD-33^T^) was isolated from a hydrothermal chimney sample from the Mid Cayman rise Beebe vent field, at 4,964 m water depth (Dalmasso et al., 2016b). The strain was grown at 75°C under anaerobic conditions, in modified Ravot medium prepared without maltose, with polysulfur (1% of polysulfide from a 0.05 M stock solution), as described elsewhere (Dalmasso et al., 2016b). Cells were grown until the mid-exponential growth phase (11 h at 0.1 MPa, 9 h at 50 MPa, and 12 h at 90 MPa) into 20 mL sterile plastic syringes incubated at 0.1, 50, and 90 MPa of hydrostatic pressure (Thermostated HP/HT incubators Top Industrie, France), from independent startup cultures grown at 50 MPa. The 3 biological replicates per pressure were incubated in the same stainless-steel pressure vessel (pressurized by pumping water into them), at the same time, together with negative controls. Cells were then harvested by centrifugation.

### Determination of Cell Numbers

Cell counts were performed on a Thoma chamber (Preciss, France; surface area: 0.0025 mm^2^, depth: 0.100 mm) and by phase contrast microscopy (Olympus BX60), to verify that the cell density was similar to that obtained at the end of the exponential growth phase during growth kinetics performed previously under exactly the same conditions.

### RNA Extraction and Purification

As soon as the cell pellets (from 20 mL cultures) were obtained, TRIzol^®^ reagent (Ambion, Life Technologies) was added to them in order to extract total RNA. DNase I treatment (Promega^®^) was then performed at 37°C for 30 min to remove genomic DNA from the isolated total RNA. Total RNA was further purified onto RNA spin columns (RNeasy Mini kit Qiagen^®^, RNA cleanup protocol), and stored at –80°C after verification of non-degradation with an Agilent 2100 Bioanalyzer (RNA 6000 Pico chip kit) and before sequencing. RNA concentrations were measured with a NanoDrop ND 1000^®^ spectrophotometer (Thermo Fisher Scientific Inc.).

### RNA-Seq

A Ribo-Zero rRNA Removal Bacteria kit (Epicenter, Madison, WI) used with manufacturer’s protocol was applied to deplete a maximum of rRNAs and enrich total RNA in mRNA. As it is often the case with *Archaea*, ribodepletion attempts were poorly efficient and the RNA-seq dataset still contained 73–94% ribosomal sequences. Ion Total RNA-Seq Kit v2 was used to make cDNA, to add barcodes, and to amplify the library. All cleanup steps were performed using Agencourt Ampure XP beads (Thermo Fisher Scientific). Each step was validated by a bioanalyzer quality control on Agilent RNA chips and/or with the “Qubit^TM^” fluorometer (Life Technologies) to determine the quality, size of fragments and quantity of produced material. RNA libraries were sequenced on P1v2 chips using the Ion S5^TM^ System (Thermo Fisher Scientific). Sequencing was completed by the GeT-Genotoul platform in Toulouse (France). The RNA-seq data have been deposited into the NIH Sequence Read Archive (SRA) under the BioProject ID PRJNA739722 (accession numbers SRX11191114-11191122).

### Bioinformatic Data Analysis

RNA-seq reads were mapped on all genes with Bowtie v2.3.1 (Langmead et al., 2009). Bowtie2 was run with default parameters and *–no-unal*. The result was converted from SAM to BAM format using Samtools v1.3.1 (Li et al., 2009). The reads counts were normalized to Transcripts Per Kilobase Million (TPM) (Supplementary Table 1). Then, TPM were mapped with Anvi’o (v7) (Eren et al., 2015) onto the *T. piezophilus* genome sequence deposited in the NCBI database under the accession number GCA_001647085.1 ASM164708v1. First, a contig database was generated (*anvi-gen-contigs-database*) with the genome sequence available on NCBI and a table containing all gene positions (*–external-gene-calls* and *–ignore-internal-stop-codons* arguments). Next, BAM files were processed individually with *anvi-init-bam* to get indexed alignment files. The latter were used to generate individual anvi’o profile to link the information available in the alignment file to the contigs databases (*anvi-profile* with required parameters and *–min-contig-length 70*). Last, all three profiles were merged with *anvi-merge* (default parameters). The command *anvi-interactive* was used to visualize the mapping results over the three conditions.

### Differential Gene Expression Analysis

Data normalization and differential gene expression analysis were done with R/bioconductor packages edgeR (Robinson et al., 2010) and DEseq2 (Love et al., 2014). For each pressure condition, the data obtained from three independent biological replicate cDNA libraries were analyzed. Principal component analysis (PCA) was used to detect potential outlier on the samples. No outlier was detected. Regarding the filtration of transcripts, EdgeR’s RLE (“Relative Log Expression”) standardization was used, with filtering of weakly expressed genes, and then correction of tests with the BH method (Benjamini-Hochberg procedure for False Discovery Rate; alpha threshold = 0.05). The DEseq2 package was used to investigate the differential expression of genes between the different pressure conditions. Transcripts that were considered statistically significant were those with an adjusted *p*-value < 1%.

### Gene Ontology Analysis

Functional annotation clustering of genes differentially expressed was carried out with the online software DAVID (Huang da et al., 2009),^1^ with default parameters.

### Motifs Binding Sites Screening

The search for SurR and TrmB-like binding motifs within *T. piezophilus* genome was carried out using in-house Perl scripts. Gene promoters were defined as the 200 pb zones upstream of the start codon of the locus tag.

## Results and Discussion

### General Response of *Thermococcus piezophilus* to Hydrostatic Pressure

**FIGURE 1:**
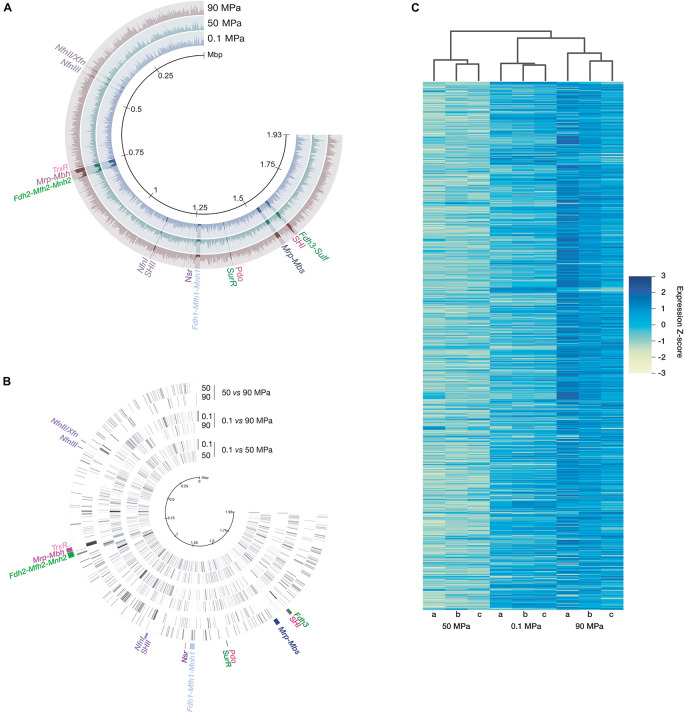
Read mapping and differential gene expression as a function of pressure variation. After normalization, the TPM were mapped on concatenated CDSs of *T. piezophilus* **(A)**. Some of the pressure-regulated genes or gene clusters are highlighted. The concentric circles represent the 3 conditions. Each ray represents a gene expression level **(B)**, ordered according to the position of the gene on the genome (indicated by the scale in the center). The logFC values were used for signal intensity and the color black was given for the maximum. Key genes of the hydrogenogenic/sulfidogenic metabolisms are displayed. These representations were made with Anvi’o (Eren et al., 2015). **(C)** Z-score hierarchical clustering heat map for visualization of the 3 biological replicates (a, b, and c) per pressure.

Nine independent biological replicates, each inoculated with a different startup culture [generated at the optimal growth pressure (50 MPa)] were incubated in high-temperature stainless steel vessels at the three selected pressures (3 × 0.1 MPa; 3 × 50 MPa; 3 × 90 MPa). Their transcriptomes were generated after RNA extraction with TRIzol^®^, then ribodepletion. After sequence filtration, there were 87,940,548 non-ribosomal usable total reads that were mapped as transcripts per kilobase million (TPM) on the 2,126 concatenated coding DNA sequence (CDS) of *T. piezophilus* (Figure 1A). After the differential expression validation step, it appeared that 1,373 of the 2,126 genes were regulated by pressure changes (Figure 1C but see Supplementary Table 2 for details) but, at most, the expression of 540 genes varied significantly when shifting from a tested pressure to another (Figure 1B and Supplementary Figure 1). At 0.1 MPa, 540 genes were overexpressed and 532 were underexpressed, while at 90 MPa, 220 genes were overexpressed and 224 genes were underexpressed compared to 50 MPa. There were more genes differentially regulated by pressure that were shared between high pressures (50 or 90 MPa) and atmospheric condition (0.1 MPa) (270 or 339 shared genes, respectively) than genes differentially regulated by pressure that were shared when comparing non-optimal pressures (0.1 or 90 MPa) to optimal growth condition (50 MPa) (109 or 125, respectively). The hierarchical clustering on z-scores highlighted a closer gene expression response between 0.1 and 90 MPa (Figure 1C).

It is therefore possible that the regulatory response to pressure variation compared to optimal growth condition (50 MPa) may be broader and more specific at atmospheric pressure than at 90 MPa, even though the growth rate is almost identical at 0.1 and 90 MPa (Dalmasso et al., 2016b).

In order to gain a better understanding of the molecular tuning that takes place into the cell, we explored more precisely (i) the expression level of the genes regulated by two-well characterized transcriptional regulators (SurR and regulators of the TrmB family), (ii) the categories of genes regulated by pressure and (iii) the function of gene clusters whose expression is impacted by pressure, with emphasis on those involved in energy conversion and conservation.

### Is Pressure a Physical Parameter Able to Modify the Transcriptional Regulation Mechanism?

The adaptive response to fluctuating environmental conditions has been less studied in *Archaea* than in other life domains (Karr, 2014). In *Thermococcales*, the regulators of the TrmB family and the SurR regulator are the two best-characterized transcriptional regulators. Regulators in the TrmB family control transcription according to sugar availability (Lee et al., 2003, 2005, 2008). These regulators bind to DNA in promoter regions to activate or repress the transcription of genes, thanks to TrmB-like binding motifs which have been published elsewhere (Gindner et al., 2014). SurR is the master regulator of primary electron flow pathways that responds directly to the presence or absence of sulfur in the environment (Lipscomb et al., 2009, 2017; Hidese et al., 2017). This ArsR-type master-regulator, which is unique to *Thermococcales* and conserved, is able to activate or repress the expression of the genes involved in the response to sulfur thanks to the presence of SurR binding motifs (GTT*n*_3_AAC or GTT*n*_3_AAC*n*_5_GTT) in their promoters (Lipscomb et al., 2009). Its sequence-specific DNA binding activity is driven by a redox-active displacement of cysteine residues within a CxxC motif. In the presence of sulfur, a disulfide bond is formed and this oxidized form of SurR inhibits its binding activity. As a result, it loses its transcriptional modulation activities which are strongly correlated with the position of its binding sites, facilitating the recruitment of transcriptional machinery or blocking its progression (Yang et al., 2010).

It is interesting to note that while this study focused on the effect of variation in hydrostatic pressure (with no change in medium composition between experiments), we observed an increase in transcripts for the three TrmB-like genes (A7C91_RS03095, A7C91_RS05650, and A7C91_RS07665) and SurR gene (A7C91_RS07565) at 0.1 MPa vs. 50 and 90 MPa (Supplementary Table 2). In order to assess the potential overall effect of this regulation, the presence of SurR and TrmB-like binding motifs was examined in *T. piezophilus* gene promoters.

TrmB-like binding motifs were detected in the promoter of only nine genes, of which only two were found to be regulated by pressure. This result seems to point to a limited role of TrmB and its targets in the response to pressure. However, due to evolutionary constraints, it is likely that the binding motifs used in our investigation in this archaeal species may be slightly different from those described in other species. In fact, these motifs were not detected in the promoter of the maltodextrin operon (MD) which is also overexpressed at 0.1 MPa vs. 50 and 90 MPa, whereas they were expected to be found there.

**FIGURE 2:**
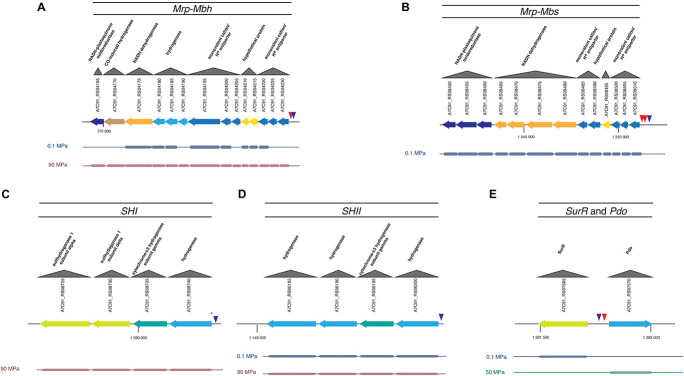
MBH, MBS, SHI, SHII, and SurR gene clusters regulated in hydrogenogenic/sulfidogenic metabolisms and their SurR binding motifs. **(A)** *Mrp-Mbh*; **(B)** *Mrp-Mbs*; **(C)** *SHI*; **(D)** *SHII*; **(E)** *SurR* and *Pdo*. Each gene is colored according to its differential expression condition (0.1 MPa in blue, 50 MPa in green, or 90 MPa in red). Triangles represent the SurR binding motifs. Red triangle is for the short binding motif (GTT*n*_3_AAC) and blue triangle is for the long one (GTT*n*_3_AAC*n*_5_GTT). The asterisk means that the motif carries a mutation. The other gene clusters regulated in hydrogenogenic/sulfidogenic metabolisms are shown in Supplementary Figure 2.

With regard to SurR, many genes under the control of this regulator have their expression modulated by pressure (Table 1; Figure 2, and Supplementary Figure 2; see next chapter). Thus, 170 promoters bearing at least one binding motif were detected among the 2,126 total CDS of *T. piezophilus*. In addition, among the 1373 CDS whose expression was regulated by pressure, 119 harbored a SurR binding motif. These values represent 8 and 8.7% of the total and overexpressed CDS, respectively, highlighting no significant increase in the expression of genes potentially under the control of SurR at different pressure conditions. However, given that 70% of genes potentially regulated by SurR are also pressure-regulated, this could be a clue of a high plasticity in adaptive response in *T. piezophilus*, but also within *Thermococcales* in general. Indeed, there is an effective variation in gene expression of SurR-regulated clusters. This could be the consequence of a pressure effect on the conformational state of SurR, modifying its binding capacity even in presence of sulfur, as has been shown for TrmB in *P. furiosus* (Krug et al., 2013). Moreover, a detailed analysis of the motif position in the promoters, revealed the presence of several potential binding sites, which, according to the BRE/TATA box, could provide some flexibility in terms of repression or activation, as for the *Mbs* cluster (Table 2). In addition, some of the genes described as being regulated by SurR in other *Thermococcales* bear mutated motifs, such as for the ones encoding SHI (Table 2), but this will need to be further investigated by an EMSA assay to see if this affects the binding affinity of SurR. One other hypothesis would be the involvement of another transcriptional regulator. This hypothesis is supported by the presence of 37 other regulators among the genes regulated by pressure (Supplementary Table 2). Their respective occurrences in terms of over-expression according to pressure, i.e., 0.1, 50, and 90 MPa, are 23, 14, and 11, respectively (Supplementary Table 2). In the light of these results, the sub-optimal condition (0.1 MPa) seems to require a higher number of regulator overexpressions than the supra-optimal growth condition (90 MPa), respectively 21 vs. 4. This correlates with the greater number of total genes regulated in these conditions (540 and 220, respectively, Supplementary Figure 1). Overall, these data support the hypothesis that atmospheric pressure requires a stronger adaptive response than higher hydrostatic pressure conditions in *T. piezophilus*.

**TABLE 1:** Variations in the expression of genes or gene clusters, known to be involved in hydrogen and sulfur metabolism.

			**Number of overexpressed coding DNA sequence (CDS)**					
**Product**	**Locus tag**	**Total CDS number**	**0.1 > 50 MPa**	**0.1 > 90 MPa**	**50 > 0.1 MPa**	**50 > 90 MPa**	**90 > 0.1 MPa**	**90 > 50 MPa**
Mrp-Mbh	A7C91_RS04230-04165	14	8	nd	nd	nd	13	14
Fdh1-Mfh1-Mnh1	A7C91_RS06945-07020	16	nd	nd	6	7	nd	nd
Fdh2-Mfh2-Mnh2	A7C91_RS04320-04240	17	8	12	nd	7	nd	nd
F_420_-reducing hydrogenase	A7C91_RS_04340-04330	3	3	3	nd	nd	nd	nd
SHI	A7C91_RS08740-08725	4	nd	nd	nd	nd	4	4
SHII	A7C91_RS06200-06185	4	4	3	nd	nd	nd	4
Mrp-Mbs	A7C91_RS08510-08450	13	12	13	nd	4	nd	nd
Nsr	A7C91_RS06865	1	nd	nd	nd	nd	nd	nd
SurR	A7C91_RS07565	1	1	1	nd	nd	nd	nd
Pdo	A7C91_RS07570	1	nd	nd	1	nd	nd	nd
Nfn I	A7C91_RS06205-6210	2	nd	nd	1	nd	2	2
Nfn II/Xfn	A7C91_RS02315-2310	2	nd	nd	2	1	1	nd
Nfn III	A7C91_RS02425-2430	2	nd	nd	1	nd	2	nd
Fdh3	A7C91_RS08745-08770	6	nd	nd	2	1	nd	nd
RNR	A7C91_RS02975	1	nd	nd	1	nd	1	nd
ATP synthase	A7C91_RS03355-03315	9	nd	nd	6	nd	7	nd
TrxR	A7C91_RS04130	1	nd	nd	1	nd	1	nd

**TABLE 2:** SurR binding motifs positions upstream from the start codon of CDS involved in the electron flow.

**Product**	**Locus tag**	**First CDS of the product**	**Motif occurrence**	**Distance (size) of the binding motif from start codon of the first CDS**		
Mrp-Mbh	A7C91_RS04230-04165	A7C91_RS04230	2	21 (short)	127 (long)	
Fdh1-Mfh1-Mnh1	A7C91_RS06945-07020	A7C91_RS06945	1	137 (short with mutation: gttaccaat)		
Fdh2-Mfh2-Mnh2	A7C91_RS04320-04240	A7C91_RS04320	1	123 (short)		
F_420_-reducing hydrogenase	A7C91_RS_04340-04330	A7C91_RS04340	1	71 (long)		
SHI	A7C91_RS08740-08725	A7C91_RS_08740	1	105 (long with mutation: gttttaacctttggtt deletion)		
SHII	A7C91_RS06200-06185	A7C91_RS06200	1	69 (long)		
Mrp-Mbs	A7C91_RS08510-08450	A7C91_RS08510	3	29 (short)	62 (short)	98 (long)
Nsr	A7C91_RS06865	A7C91_RS06865	1	17 (short)		
SurR	A7C91_RS07565	A7C91_RS07565	2	49 (long)	103 (short)	
Pdo	A7C91_RS07570	A7C91_RS07570	2	23 (short)	69 (short)	
Nfn I	A7C91_RS06205-6210	A7C91_RS06205	1	72 (long)		
Nfn II/Xfn	A7C91_RS02315-2310	A7C91_RS02315	2	44 (short)	72 (short)	
Nfn III	A7C91_RS02425-2430	A7C91_RS02425	1	42 (short)		
Fdh3	A7C91_RS08745-08770	A7C91_RS04340	1	71 (long)		
RNR	A7C91_RS02975	A7C91_RS02975	0	/		
ATP synthase	A7C91_RS03355-03315	A7C91_RS03355	0	/		
TrxR	A7C91_RS04130	A7C91_RS04130	0	/		
Short: GTT*n*3AAC, long: GTT*n*3AAC*n*5GTT						

### Categories of Genes Regulated by Pressure

To uncover the molecular mechanisms behind the pressure adaptive responses, the differentially expressed genes (DEG) were analyzed with the functional annotation tool DAVID which allows identifying functional categories and ranking them based on gene-enrichments in annotation terms (EASE score) (Huang da et al., 2009; Supplementary Table 3). Gene clusters characterized by a gene-enrichment (in this instance having an EASE score superior to 1) were analyzed in details. Results are presented in Supplementary Figure 1. The broadest functional response was predicted for 90 MPa vs. 0.1 MPa with five different classes of genes that were enriched (iron, hydrogen ion transport, ligase, ribosomal protein and nucleotidyltransferase) vs. one or two for the other conditions. These results show that the number of regulated genes can be twice as high in one comparison of conditions as in another without necessarily being more functionally diverse (540 vs. 220 overexpressed genes at 0.1 and 90 MPa, respectively against 50 MPa). As observed in other *Thermococcus* species, the adaptive response to pressure variations in *Thermococcales* appears to be more balanced, global and integrative than a classical response to a physical stress (Vannier et al., 2015; Michoud and Jebbar, 2016).

### Functions of the Gene Clusters Regulated by Pressure

#### Main Functions Impacted by Pressure

The vast majority of gene expression variation was related to a global metabolic response, as described for *T. barophilus* and *P. yayanosii* (Vannier et al., 2015; Michoud and Jebbar, 2016). Variations in the level of transcription occurred globally at the level of gene clusters (Figure 1A). *T. piezophilus* appeared to adapt to pressure changes by modulating amino acid and vitamin synthesis, production of potential compatibles solutes, and regulating the chemotaxis, S-layer and CRISPR-Cas systems. All these results are described in details in Supplementary Data 1. In summary, the strain does not possess the genetic potential to synthesize the three aromatic amino acids L-phenylalanine, L-tyrosine, and L-tryptophan, and underexpresses genes for the synthesis of a.a histidine while overexpressing genes encoding different families of transporters of amino acids, of oligopeptides and of dipeptides under non-optimal conditions. In the same fashion, few genes of thiamine and biotin pathways were underexpressed at 0.1 and 90 MPa while some vitamin transporters were overexpressed at 90 MPa. These observations suggest that the import of certain amino acids and vitamins may be preferred over *de novo* synthesis under non-optimal pressures. Furthermore, overexpression of glutamate biosynthesis genes was observed at 0.1 MPa, suggesting an overproduction of this metabolite at low pressures, possibly to help support macromolecule conformation and function. In addition, a large majority of the genes encoding the chemotaxis pathway were transcriptionally overexpressed at 50 MPa, and even more so at 90 MPa, compared to 0.1 MPa (Supplementary Figure 3), as described in Supplementary Data 1. Finally, two genes coding for protein subunits of the S-layer and the gene clusters of the CRISPR-*cas* type I-B and of the CRISPR-*cas* type III-A systems were overexpressed at 0.1 MPa, as detailed in Supplementary Data 1. These results are consistent with those reported for other piezophilic *Thermococales* or other piezophilic microorganisms (Amrani et al., 2014; Vannier et al., 2015; Michoud and Jebbar, 2016), hence they are mainly detailed in Supplementary Data 1.

Hydrogenases and energy conversion circuits that were transcriptionally modulated by pressure were explored in detail in this article. They are developed below.

#### Effect of Pressure on Hydrogenases and Energy Conversion Circuits

##### Effect of Hydrostatic Pressure on Membrane-Bound Hydrogenases MBH and MBS

**FIGURE 3:**
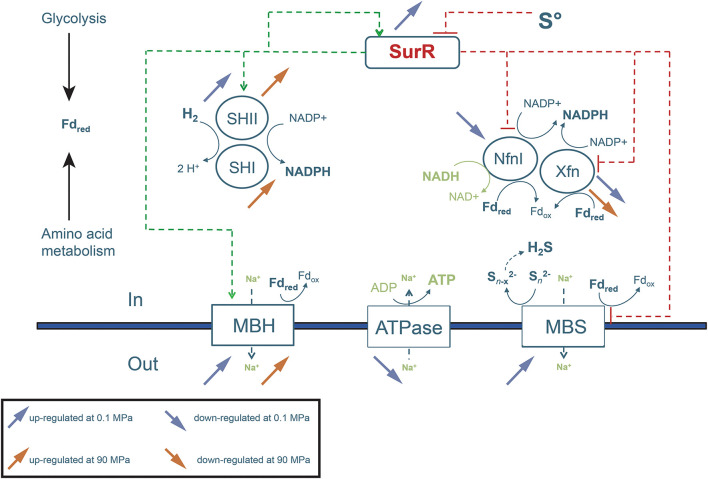
Schematic representation of the main transcriptional metabolic regulations observed at sub-optimal (0.1 MPa) and supra-optimal (90 MPa) pressures for growth. This figure is centered on the general metabolism and focuses notably on hydrogenases and energy conversion processes and on their transcriptional regulator SurR, in the context of adaptive response to hydrostatic pressure. Blue and red arrows represent the overexpression (up direction) and subexpression (down direction) of genes at sub-optimal (0.1 MPa), and supra-optimal (90 MPa) pressures for growth, respectively, compared to the optimal pressure for growth (50 MPa).

Members of the *Thermococcales* order grow heterotrophically by fermentation of peptides or sugars used as carbon and energy sources. This leads to the production of CO_2_, acetate, and gas, whose composition, respectively H_2_S or H_2_, depends in particular on the presence or absence of elemental sulfur in the medium (Verhees et al., 2003; Sakuraba et al., 2004). The metabolism generates reduced ferredoxins that are used by membrane-bound oxidoreductases to create an ion gradient with the extracellular environment (Kengen et al., 1996; Verhees et al., 2003; Sakuraba et al., 2004). This ion gradient can further be used by an ATP synthase to produce ATP (Figure 3; Pisa et al., 2007). In *Thermococcales* in general and *T. piezophilus* in particular, two main systems of membrane bound oxidoreductases exist (Schut et al., 2013). The first one is the simple respiratory system MBH (membrane-bound hydrogenase) that is generally activated in the absence of S° (Sapra et al., 2003) and links the oxidation of reduced ferredoxin generated during glycolysis to the formation of H_2_ (Kanai et al., 2011; Schut et al., 2012). When sulfur is provided in the medium, a second type of membrane oxidoreductase, is active: the MBS (Membrane-bound sulfane reductase) system (Wu et al., 2018), highly homologous to MBH (13 vs. 14-genes cluster). Recent studies have shown the generation of H_2_S from spontaneous breakdown of tri- and disulfides after their release from MBS (Wu et al., 2018; Yu et al., 2020). This orientation toward the hydrogenogenic or/and the sulfidogenic pathway is orchestrated by the transcriptional sulfur-response regulator SurR (Lipscomb et al., 2009, 2017; Yang et al., 2010; Hidese et al., 2017).

Our transcriptomic study has shown that the level of expression of both MBH and MBS gene clusters is modulated by pressure (Table 1). This result is surprising, as *T. piezophilus* has been grown with polysulfur in all these experiments. Indeed, numerous studies on *Thermococcales* carried out at atmospheric pressure have shown that the genes coding for the MBS complex have their level of transcription increased when sulfur is present in the culture medium. On the contrary, it has been shown that the level of expression of the genes coding for the MBH complex decreases sharply in the presence of sulfur (still at atmospheric pressure) (Chou et al., 2007; Jager et al., 2014). Here, our results show that the expression level of the *Mbh* gene cluster varies despite the presence of sulfur. At the sub-optimal pressure of 0.1 MPa, the gene clusters of the MBS and MBH complexes are both overexpressed (Table 1 and Figures 2, 3), compared to 50 MPa. The genes of the *Mbh* cluster were considerably overexpressed at the supra-optimal pressure of 90 MPa compared to 50 and 0.1 MPa. It was also slightly more transcribed at 0.1 MPa compared to 50 MPa (Table 1 and Figures 2, 3). This suggests a pressure-effect overtaking a sulfur-effect on the regulation of gene expression.

##### Effect of Hydrostatic Pressure on Cytosolic Hydrogenases, Oxidoreductases, and Glycolytic Enzymes

Other enzymes involved in these H_2_/sulfur metabolic pathways (Figure 3) include the cytosolic hydrogenases and oxidoreductases SHI/II, NfnI/II/III, Pdo, and TrxR, which have been shown to be under the regulation of SurR (Lipscomb et al., 2017). SHI and SHII are soluble hydrogenases, that could couple the synthesis of protons from an H_2_ uptake to the regeneration of NADPH from NADP + (Schut et al., 2013). The ferredoxin:NADP + oxidoreductases (Nfn) of *Thermococcales* are involved in the electron transfer from ferredoxin to NADP +, under high concentrations of H_2_ (Verhaart et al., 2010). The subsequent NADPH can be then reoxidized by a glutamate dehydrogenase and an alanine amino-transferase in the alanine biosynthetic pathway. Pdo is another glutaredoxin-like protein disulfide oxidoreductase whose roles are not totally understood. It is notably regulated by SurR and steps in the overall cellular redox balance and in the hydrogen metabolism. It could receive electrons from NADPH through a thioredoxin reductase (TrxR), and then serve as an electron carrier itself for ribonucleotide reductase (RNR), allowing links to deoxyribonucleotide synthesis (Schut et al., 2013; Demmer et al., 2015).

Here, an important overexpression of the gene cluster encoding the soluble hydrogenase SHII was observed at 0.1 and 90 MPa (Figure 2) compared to 50 MPa. This overexpression was more pronounced at 0.1 MPa (Table 1). Genes encoding the cytoplasmic SHI hydrogenase subunits were overexpressed but only at 90 MPa compared to 50 and 0.1 MPa (Figure 2). These soluble hydrogenases could thus be mobilized under stressful pressure conditions to regenerate NADPH. An overexpression of the NADH-dependent ferredoxin:NADP + oxidoreductases (Nfn) was observed at 50 and 90 MPa, but not at atmospheric pressure. As with “*Thermococcus onnurineus*,” *T. piezophilus* has 2 genes coding for homologs of the S and L subunits of NfnI (A7C91_RS06205-6210) of *P. furiosus* (Nguyen et al., 2017), and also possesses NfnII (A7C91_RS02315-2310) and NfnIII (A7C91_RS02425-2430). *NfnI* was overexpressed at 90 MPa vs. 50 MPa, while *NfnII* was overexpressed at 50 MPa (vs. 0.1 MPa). *NfnIII* was overexpressed at 90 MPa vs. 0.1 MPa. In addition, genes encoding a glutaredoxin-like protein (A7C91_RS07570) and the disulfide oxidoreductase Pdo (A7C91_RS07570) were overexpressed at 50 MPa. Genes coding for the thioredoxin reductase TrxR (A5C91_RS04130) and for the ribonucleotide reductase RNR (AC91_RS02975) were overexpressed at 50 and 90 MPa vs. 0.1 MPa. Altogether, these observations tend to indicate that under stressful conditions, H_2_ production (with Mbh) is activated. In addition, at 0.1 MPa, the *Mbs*, and *Mbh* gene clusters are overexpressed, suggesting a cumulative effect to cope with pressure. Soundly, several genes coding for glycolytic enzymes, a pathway which regenerates reduced ferredoxin, were overexpressed at the reference pressure of 50 MPa [i.e., ADP-dependent glucokinase (A7C91_RS07115), phosphoglycerate kinase (A7C91_RS00540) and glyceraldehyde-3-phosphate dehydrogenase (A7C91_RS09215)], and also at 90 MPa [glyceraldehyde-3-phosphate dehydrogenase, glyceraldehyde-3-phosphate ferredoxin oxidoreductase (A7C91_RS04780) (GAPOR)].

##### Effect of Hydrostatic Pressure on Other Mrp-Mbh Oxidoreductases

In *T. piezophilus*, other types of *Mrp-Mbh* oxidoreductases clusters were also regulated by pressure. These clusters code for formate dehydrogenases (FDH) which are found only in some of the *Thermococcales*, such as *P. yayanosii* and “*T. onnurineus”* (Schut et al., 2013). Thus, three formate dehydrogenase gene clusters were found in *T. piezophilus* genome, similar to those of “*T. onnurineus*,” namely the membrane-bound hydrogenase clusters *Fdh1-Mfh1-Mnh1* (A7C91_RS06945-07020) and *Fdh2-Mfh2-Mnh2* (A7C91_RS04320-04240), and the cytoplasmic hydrogenase cluster *Fdh3* (A7C91_RS08745-08770). The synteny of these clusters is conserved between both organisms. The presence of the *Fdh2-Mfh2-Mnh2* gene cluster together with the one encoding a formate transporter suggests that *T. piezophilus* could metabolize formate. This was not observed experimentally at atmospheric pressure (Dalmasso et al., 2016a), likely because growth on formate was tested at 75°C, under conditions where the reaction was probably not exergonic (Kim et al., 2010).

At 50 MPa, genes of the clusters *Fdh1-Mfh1-Mnh1* and *Fdh3* were overexpressed, as well as the associated ATP synthase clusters. Genes of the cluster *Fdh2-Mfh2-Mnh2* were also more transcribed at 50 MPa and even more again at 0.1 MPa. This might indicate that growth on formate could be stimulated under optimal and sub-optimal pressure conditions.

A cytoplasmic homolog of the F_420_-reducing hydrogenase (A7C91_RS04340-04330) present in methanogenic archaea (*frhAGB*-encoding hydrogenase) whose expression is linked to various functions in *Thermococcus* sp., including carbon monoxide metabolism, chemotactic signal transduction and *Fdh3* regulation (Jeon et al., 2015; Lee et al., 2017), was also overexpressed at 0.1 MPa compared to 50 and to 90 MPa. This hydrogenase is involved in various cellular processes in “*T. onnurineus*,” which could also be the case in *T. piezophilus*. This observation is also consistent with the fact that the cell undergoes a broad metabolic change to cope with pressure variations.

##### Effect of Pressure on SurR-Regulated Genes: A Synthesis

In summary, with the exception of genes of the *Mrp-Mbs* cluster, genes encoding enzymes which are known to be under SurR negative control, namely *Pdo* and *Nfn*, were all overexpressed at the reference pressure of 50 MPa vs. 0.1 MPa (in presence of sulfur). Interestingly, the genes and gene clusters that were under SurR positive control were overexpressed at supra- and sometimes sub-optimal pressures for growth (see above). This suggests that, in *T. piezophilus*, the expression patterns of genes known to be under the control of the SurR regulon appear to follow a classical response/behavior to sulfur only under optimal pressure conditions. Sub-optimal pressures and, to a lesser extent, supra-optimal pressures are likely to influence the expression of these genes in a new way of regulation (Figure 3). As an illustration, a cooperation between the membrane complexes MBH and MBS appears to occur at atmospheric pressure regardless of the presence of sulfur.

## Conclusion

This study presents the transcriptional adaptive strategy implemented by *T. piezophilus*, the piezophilic microorganism with the widest range of tolerance to HHP (0.1–130 MPa) known to date. The overall differential gene expression revealed by RNA-seq confirms the results previously obtained with *T. barophilus* and *P. yayanosii* (Vannier et al., 2015; Michoud and Jebbar, 2016) that there is an overall metabolic change rather than a classical stress response. A thorough knowledge of the process of energy-conservation and its regulation in other *Thermococcales* models, has enabled us to highlight the involvement of these processes in the adaptive response, at least in the regulation of the expression of genes coding for the proteins involved in this process. These results are changing our knowledge of the mechanism of regulation of this energy-conservation process under conditions of non-optimal pressures and of the role of SurR under these conditions.

## Data Availability Statement

The datasets presented in this study can be found in online repositories. The names of the repository/repositories and accession number(s) can be found in the article/Supplementary Material.

## Author Contributions

KA and CD designed the experiment. CD and MG carried out the experiments. YM and DC performed the bioinformatics analyses. KA, YM, and JH interpreted the data and wrote the manuscript. PO, MJ, and ZS helped supervise the project. All authors reviewed the manuscript.

## Conflict of Interest

The authors declare that the research was conducted in the absence of any commercial or financial relationships that could be construed as a potential conflict of interest.

## Publisher’s Note

All claims expressed in this article are solely those of the authors and do not necessarily represent those of their affiliated organizations, or those of the publisher, the editors and the reviewers. Any product that may be evaluated in this article, or claim that may be made by its manufacturer, is not guaranteed or endorsed by the publisher.

## Footnotes

1
https://david.ncifcrf.gov/summary.jsp

## References

[B1] Amrani A. Bergon A. Holota H. Tamburini C. Garel M. Ollivier B. (2014). Transcriptomics reveal several gene expression patterns in the piezophile *Desulfovibrio hydrothermalis* in response to hydrostatic pressure. *PLoS One* 9:e106831. 10.1371/journal.pone.0106831 25215865 PMC4162548

[B2] Cario A. Grossi V. Schaeffer P. Oger P. M. (2015a). Membrane homeoviscous adaptation in the piezo-hyperthermophilic archaeon Thermococcus barophilus. *Front. Microbiol.* 6:1152. 10.3389/fmicb.2015.01152 26539180 PMC4612709

[B3] Cario A. Jebbar M. Thiel A. Kervarec N. Oger P. M. (2016). Molecular chaperone accumulation as a function of stress evidences adaptation to high hydrostatic pressure in the piezophilic archaeon Thermococcus barophilus. *Sci. Rep.* 6:29483. 27378270 10.1038/srep29483 PMC4932500

[B4] Cario A. Lormieres F. Xiang X. Oger P. (2015b). High hydrostatic pressure increases amino acid requirements in the piezo-hyperthermophilic archaeon Thermococcus barophilus. *Res. Microbiol.* 166 710–716. 10.1016/j.resmic.2015.07.004 26226334

[B5] Chou C. J. Shockley K. R. Conners S. B. Lewis D. L. Comfort D. A. Adams M. W. (2007). Impact of substrate glycoside linkage and elemental sulfur on bioenergetics of and hydrogen production by the hyperthermophilic archaeon Pyrococcus furiosus. *Appl. Environ. Microbiol.* 73 6842–6853. 10.1128/aem.00597-07 17827328 PMC2074980

[B6] Dalmasso C. Oger P. Courtine D. Georges M. Takai K. Maignien L. (2016a). Complete genome sequence of the hyperthermophilic and piezophilic archeon thermococcus piezophilus CDGST, able to grow under extreme hydrostatic pressures. *Genome Announc.* 4:e00610–16. 10.1128/genomeA.00610-16 27417831 PMC4945791

[B7] Dalmasso C. Oger P. Selva G. Courtine D. Lharidon S. Garlaschelli A. (2016b). Thermococcus piezophilus sp. nov., a novel hyperthermophilic and piezophilic archaeon with a broad pressure range for growth, isolated from a deepest hydrothermal vent at the Mid-Cayman Rise. *Syst. Appl. Microbiol.* 39 440–444. 10.1016/j.syapm.2016.08.003 27638197

[B8] Demmer J. K. Huang H. Wang S. Demmer U. Thauer R. K. Ermler U. (2015). Insights into flavin-based electron bifurcation via the NADH-dependent Reduced Ferredoxin:NADP oxidoreductase structure. *J. Biol. Chem.* 290 21985–21995. 10.1074/jbc.M115.656520 26139605 PMC4571952

[B9] Eren A. M. Esen O. C. Quince C. Vineis J. H. Morrison H. G. Sogin M. L. (2015). Anvi’o: an advanced analysis and visualization platform for ’omics data. *PeerJ* 3:e1319. 10.7717/peerj.1319 26500826 PMC4614810

[B10] Gindner A. Hausner W. Thomm M. (2014). The TrmB family: a versatile group of transcriptional regulators in Archaea. *Extremophiles* 18 925–936. 10.1007/s00792-014-0677-2 25116054 PMC4158304

[B11] Hidese R. Yamashita K. Kawazuma K. Kanai T. Atomi H. Imanaka T. (2017). Gene regulation of two ferredoxin:NADP(+) oxidoreductases by the redox-responsive regulator SurR in Thermococcus kodakarensis. *Extremophiles* 21 903–917. 10.1007/s00792-017-0952-0 28688056

[B12] Huang da W. Sherman B. T. Lempicki R. A. (2009). Systematic and integrative analysis of large gene lists using DAVID bioinformatics resources. *Nat. Protoc.* 4 44–57. 10.1038/nprot.2008.211 19131956

[B13] Jager D. Forstner K. U. Sharma C. M. Santangelo T. J. Reeve J. N. (2014). Primary transcriptome map of the hyperthermophilic archaeon Thermococcus kodakarensis. *BMC Genomic.* 15:684. 10.1186/1471-2164-15-684 25127548 PMC4247193

[B14] Jannasch H. W. Taylor C. D. (1984). Deep-sea microbiology. *Ann. Rev. Microbiol.* 38 487–514. 6437324 10.1146/annurev.mi.38.100184.002415

[B15] Jebbar M. Franzetti B. Girard E. Oger P. (2015). Microbial diversity and adaptation to high hydrostatic pressure in deep-sea hydrothermal vents prokaryotes. *Extremophiles* 19 721–740. 10.1007/s00792-015-0760-3 26101015

[B16] Jeon J. H. Lim J. K. Kim M.-S. Yang T.-J. Lee S.-H. Bae S. S. (2015). Characterization of the frhAGB-encoding hydrogenase from a non-methanogenic hyperthermophilic archaeon. *Extremophiles* 19 109–118. 10.1007/s00792-014-0689-y 25142159

[B17] Kanai T. Matsuoka R. Beppu H. Nakajima A. Okada Y. Atomi H. (2011). Distinct physiological roles of the three [NiFe]-hydrogenase orthologs in the hyperthermophilic archaeon Thermococcus kodakarensis. *J. Bacteriol.* 193 3109–3116. 10.1128/JB.01072-10 21515783 PMC3133214

[B18] Karr E. A. (2014). “Chapter Three - Transcription Regulation in the Third Domain,” in *Advances in Applied Microbiology*, eds Sariaslani S. Gadd G. M. (Cambridge: Academic Press), 101–133. 10.1016/b978-0-12-800259-9.00003-2 25131401

[B19] Kengen S. M. Stams A. J. M. De Vos W. M. (1996). Sugar metabolism of hyperthermophiles. *FEMS Microbiol. Rev.* 18 119–137. 10.1111/j.1574-6976.1996.tb00231.x

[B20] Kim Y. J. Lee H. S. Kim E. S. Bae S. S. Lim J. K. Matsumi R. (2010). Formate-driven growth coupled with H_2_ production. *Nature* 467:352. 10.1038/nature09375 20844539

[B21] Krug M. Lee S.-J. Boos W. Diederichs K. Welte W. (2013). The three-dimensional structure of TrmB, a transcriptional regulator of dual function in the hyperthermophilic archaeon Pyrococcus furiosus in complex with sucrose. *Protein Sci.* 22 800–808. 10.1002/pro.2263 23576322 PMC3690719

[B22] Langmead B. Trapnell C. Pop M. Salzberg S. L. (2009). Ultrafast and memory-efficient alignment of short DNA sequences to the human genome. *Genome Biol.* 10:R25. 10.1186/gb-2009-10-3-r25 19261174 PMC2690996

[B23] Lee S. H. Kim M. S. Kim Y. J. Kim T. W. Kang S. G. Lee H. S. (2017). Transcriptomic profiling and its implications for the H2 production of a non-methanogen deficient in the frhAGB-encoding hydrogenase. *Appl. Microbiol. Biotechnol.* 101 5081–5088. 10.1007/s00253-017-8234-4 28341885

[B24] Lee S.-J. Engelmann A. Horlacher R. Qu Q. Vierke G. Hebbeln C. (2003). TrmB, a sugar-specific transcriptional regulator of the trehalose/maltose ABC transporter from the hyperthermophilic archaeon Thermococcus litoralis. *J. Biol. Chem.* 278 983–990. 10.1074/jbc.m210236200 12426307

[B25] Lee S.-J. Moulakakis C. Koning S. M. Hausner W. Thomm M. Boos W. (2005). TrmB, a sugar sensing regulator of ABC transporter genes in Pyrococcus furiosus exhibits dual promoter specificity and is controlled by different inducers. *Mol. Microbiol.* 57 1797–1807. 16135241 10.1111/j.1365-2958.2005.04804.x

[B26] Lee S. J. Surma M. Hausner W. Thomm M. Boos W. (2008). The role of TrmB and TrmB-like transcriptional regulators for sugar transport and metabolism in the hyperthermophilic archaeon Pyrococcus furiosus. *Arch. Microbiol.* 190:247. 18470695 10.1007/s00203-008-0378-2

[B27] Li H. Handsaker B. Wysoker A. Fennell T. Ruan J. Homer N. (2009). The Sequence Alignment/Map format and SAMtools. *Bioinformatics* 25 2078–2079. 10.1093/bioinformatics/btp352 19505943 PMC2723002

[B28] Lipscomb G. L. Keese A. M. Cowart D. M. Schut G. J. Thomm M. Adams M. W. (2009). SurR: a transcriptional activator and repressor controlling hydrogen and elemental sulphur metabolism in Pyrococcus furiosus. *Mol. Microbiol.* 71 332–349. 10.1111/j.1365-2958.2008.06525.x 19017274 PMC2745277

[B29] Lipscomb G. L. Schut G. J. Scott R. A. Adams M. W. W. (2017). SurR is a master regulator of the primary electron flow pathways in the order Thermococcales. *Mol. Microbiol.* 104 869–881. 10.1111/mmi.13668 28295726

[B30] Love M. I. Huber W. Anders S. (2014). Moderated estimation of fold change and dispersion for RNA-seq data with DESeq2. *Genome Biol.* 15:550. 10.1186/s13059-014-0550-8 25516281 PMC4302049

[B31] Martinez N. Michoud G. Cario A. Ollivier J. Franzetti B. Jebbar M. (2016). High protein flexibility and reduced hydration water dynamics are key pressure adaptive strategies in prokaryotes. *Sci. Rep.* 6:32816. 10.1038/srep32816 27595789 PMC5011708

[B32] Michoud G. Jebbar M. (2016). High hydrostatic pressure adaptive strategies in an obligate piezophile Pyrococcus yayanosii. *Sci. Rep.* 6:27289. 10.1038/srep27289 27250364 PMC4890121

[B33] Nguyen D. M. N. Schut G. J. Zadvornyy O. A. Tokmina-Lukaszewska M. Poudel S. Lipscomb G. L. (2017). Two functionally distinct NADP(+)-dependent ferredoxin oxidoreductases maintain the primary redox balance of Pyrococcus furiosus. *J. Biol. Chem.* 292 14603–14616. 10.1074/jbc.M117.794172 28705933 PMC5582851

[B34] Oger P. M. Jebbar M. (2010). The many ways of coping with pressure. *Res. Microbiol.* 161 799–809. 10.1016/j.resmic.2010.09.017 21035541

[B35] Picard A. Daniel I. (2013). Pressure as an environmental parameter for microbial life–a review. *Biophys. Chem.* 183 30–41. 10.1016/j.bpc.2013.06.019 23891571

[B36] Pisa K. Y. Huber H. Thomm M. Müller V. (2007). A sodium ion-dependent A1AO ATP synthase from the hyperthermophilic archaeon Pyrococcus furiosus. *Febs J.* 274 3928–3938. 10.1111/j.1742-4658.2007.05925.x 17614964

[B37] Robinson M. D. Mccarthy D. J. Smyth G. K. (2010). edgeR: a Bioconductor package for differential expression analysis of digital gene expression data. *Bioinformatics* 26 139–140. 10.1093/bioinformatics/btp616 19910308 PMC2796818

[B38] Sakuraba H. Goda S. Ohshima T. (2004). Unique sugar metabolism and novel enzymes of hyperthermophilic archaea. *Chem. Rec.* 3 281–287. 10.1002/tcr.10066 14762828

[B39] Sapra R. Bagramyan K. Adams M. W. (2003). A simple energy-conserving system: proton reduction coupled to proton translocation. *Proc. Natl. Acad. Sci. USA* 100 7545–7550. 10.1073/pnas.1331436100 12792025 PMC164623

[B40] Schut G. J. Boyd E. S. Peters J. W. Adams M. W. (2013). The modular respiratory complexes involved in hydrogen and sulfur metabolism by heterotrophic hyperthermophilic archaea and their evolutionary implications. *FEMS Microbiol. Rev.* 37 182–203. 10.1111/j.1574-6976.2012.00346.x 22713092

[B41] Schut G. J. Nixon W. J. Lipscomb G. L. Scott R. A. Adams M. W. (2012). Mutational analyses of the enzymes involved in the metabolism of hydrogen by the hyperthermophilic archaeon pyrococcus furiosus. *Front. Microbiol.* 3:163. 10.3389/fmicb.2012.00163 22557999 PMC3341082

[B42] Siliakus M. F. Van Der Oost J. Kengen S. W. M. (2017). Adaptations of archaeal and bacterial membranes to variations in temperature, pH and pressure. *Extremophiles* 21 651–670. 10.1007/s00792-017-0939-x 28508135 PMC5487899

[B43] Simonato F. Campanaro S. Lauro F. M. Vezzi A. Dangelo M. Vitulo N. (2006). Piezophilic adaptation: a genomic point of view. *J. Biotechnol.* 126 11–25. 10.1016/j.jbiotec.2006.03.038 16780980

[B44] Vannier P. Michoud G. Oger P. Marteinsson V. Jebbar M. (2015). Genome expression of Thermococcus barophilus and Thermococcus kodakarensis in response to different hydrostatic pressure conditions. *Res. Microbiol.* 166 717–725. 10.1016/j.resmic.2015.07.006 26239966

[B45] Verhaart M. R. Bielen A. A. Van Der Oost J. Stams A. J. Kengen S. W. (2010). Hydrogen production by hyperthermophilic and extremely thermophilic bacteria and archaea: mechanisms for reductant disposal. *Environ. Technol.* 31 993–1003. 10.1080/09593331003710244 20662387

[B46] Verhees C. H. Kengen S. W. Tuininga J. E. Schut G. J. Adams M. W. De Vos W. M. (2003). The unique features of glycolytic pathways in Archaea. *Biochem. J.* 375 231–246. 10.1042/bj20021472 12921536 PMC1223704

[B47] Wu C. H. Schut G. J. Poole F. L. II Haja D. K. Adams M. W. W. (2018). Characterization of membrane-bound sulfane reductase: A missing link in the evolution of modern day respiratory complexes. *J. Biol. Chem.* 293 16687–16696. 10.1074/jbc.ra118.005092 30181217 PMC6204914

[B48] Yang H. Lipscomb G. L. Keese A. M. Schut G. J. Thomm M. Adams M. W. (2010). SurR regulates hydrogen production in Pyrococcus furiosus by a sulfur-dependent redox switch. *Mol. Microbiol.* 77 1111–1122. 20598080 10.1111/j.1365-2958.2010.07275.x PMC2975895

[B49] Yayanos A. A. (1995). Microbiology to 10,500 meters in the deep-sea. *Ann. Rev. Microbiol.* 49 777–805. 10.1146/annurev.mi.49.100195.004021 8561479

[B50] Yu H. Haja D. K. Schut G. J. Wu C.-H. Meng X. Zhao G. (2020). Structure of the respiratory MBS complex reveals iron-sulfur cluster catalyzed sulfane sulfur reduction in ancient life. *Nat. Commun.* 11:5953. 33230146 10.1038/s41467-020-19697-7 PMC7684303

