# Supplementary material for: The Piezo-Hyperthermophilic Archaeon Thermococcus piezophilus Regulates Its Energy Efficiency System to Cope With Large Hydrostatic Pressure Variations
Source: Front Microbiol. 2021 Nov 3;12:730231. doi: 10.3389/fmicb.2021.730231 (PMC8595942; doi:10.3389/fmicb.2021.730231)
Supplement: Supplementary file 1 [file Data_Sheet_1.docx]

**Supplementary Information 1**

**The piezo-hyperthermophilic archaeon *Thermococcus piezophilus* regulates its energy efficiency system to cope with large hydrostatic pressure variations**

Yann Moalic^1,2^, Jordan Hartunians^1,2^, Cécile Dalmasso^1,2^, Damien Courtine^1,2^, Myriam Georges^1,2^, Philippe Oger^3^, Zongze Shao^4,2^, Mohamed Jebbar^1,2^ & Karine Alain^1,2^

^1^ Univ Brest, CNRS, Ifremer, Laboratoire de Microbiologie des Environnements Extrêmes LM2E, UMR 6197, IUEM, Rue Dumont d’Urville, F-29280 Plouzané, France

^2^ IRP 1211 MicrobSea, Sino-French Laboratory of Deep-Sea Microbiology, LM2E (Plouzané, France)-KLAMBR (Xiamen, China)

^3^ Université de Lyon, INSA Lyon, CNRS UMR 5240, 11 Avenue Jean Capelle, F-69621 Villeurbanne, France.

^4^ Key Laboratory of Marine Biogenetic Resources, the Third Institute of Oceanography SOA, Xiamen, Fujian 361005, PR China.

**Amino acid and vitamin synthesis**

Based on the MicroCyc collection of microbial Pathway/Genome Databases (PGDBs) implemented onto the MicroScope platform, the genome of *T. piezophilus* possesses complete circuits for the production of alanine, asparagine, aspartate, glutamate, glutamine, glycine and histidine. To the best of our knowledge, at the time of writing, the synthesis pathways of other amino acids do not seem complete. The biosynthesis of aromatic amino acid is absent or downregulated by supra-optimal pressures in several piezotolerant and piezophilic taxa, obviously because of its energetic cost (i.e. the eukaryote *Saccharomyces cerevisiae*, the bacterium *Desulfovibrio hydrothermalis*, the archaea *Pyrococcus yayanosii*) (Abe and Horikoshi, 2000; Amrani et al., 2014; Michoud and Jebbar, 2016). In *T. piezophilus*, the biosynthesis pathway for chorismate, an intermediate in the production of the three aromatic amino acids L-phenylalanine, L-tyrosine and L-tryptophan, is incomplete, and most of the necessary enzymes are missing. At the reference pressure of 50 MPa, and compared to the sub-optimal pressure 0.1 MPa, genes encoding the following are overexpressed: proteins leading to the aromatic amino acid histidine, ATP phosphoribosyltransferase regulatory subunit and ATP phosphoribosyltransferase (A7C91_RS10575-80, *HisG*), histidinol dehydrogenase (A7C91_RS10585, *HisD*), imidazole glycerol phosphate synthase (A7C91_RS10595, *HisH*) and 1-(5-phosphoribosyl)-5-[(5-phosphoribosylamino)methylideneamino] imidazole-4-carboxamide isomerase (A7C91_RS10600, *HisA*). As detailed below, genes involved in glutamate synthesis are also overexpressed with decreasing pressures. An aspartate production enzyme (aspartate aminotransferase, A7C91_RS06230) is overexpressed at atmospheric pressure over optimal and supra-optimal pressures 50 MPa and 90 MPa. Genes of the glycine biosynthesis pathway II (key enzyme: glycine cleavage system protein H, locus A7C91_RS02290) and of the glycine biosynthesis pathway III (key enzyme: alanine glyoxylate transaminase, locus A7C91_RS00320) are slightly overexpressed at 50 MPa compared to atmospheric pressure and 90 MPa, respectively. By contrast, genes of the glycine biosynthesis pathway I (key enzyme: serine hydroxymethyltransferase, locus A7C91_RS10280) have upregulated expression at 0.1 MPa.

Transcription of genes coding for different families of transporters of amino acids, of oligopeptides and of dipeptides (*e.g*. peptide/oligopeptide ABC transporter ATP-binding proteins (A7C91_RS03250-70), oligopeptide transporter OPT family (A7C91_RS03135), amino-acid permease (A7C91_RS03785)), increases, sometimes strongly, under sub-optimal pressure (0.1 MPa). We can notably observe strong signals of overexpression and a wide range of overexpressed transporters at atmospheric pressure (31 genes). These observations suggest that import of amino acids/peptides is preferred over *de novo* synthesis under non-optimal pressures.

In the same fashion, few genes of vitamins biosynthetic pathways (thiamine and biotin) are overexpressed at 50 MPa compared to 0.1 and 90 MPa. They include the biotin synthase (A7C91_RS02560 and A7C91_RS04090) and the thiamine biosynthesis protein ThiS (A7C91_RS03040 and A7C91_RS07215). As vitamin biosynthesis has a high energy cost in prokaryotes, we may imagine that import should be preferred to *de novo* synthesis under non-optimal pressure conditions. We also observed, at 90 MPa, an overexpression of genes coding for transporters of other vitamins (B12 vitamin, A7C91_RS05250).

**Compatible solute biosynthesis**

Enzyme annotation and function prediction carried out with MicroCyc indicate that *T. piezophilus* possesses in its genome the complete pathways for the synthesis of the 3 following compounds: glutamine, glutamate and di-*myo*-inositol-phosphate (DIP). These compounds can all act potentially as compatible solutes (Empadinhas and da Costa, 2011; Gregory and Boyd, 2021) and help maintain cellular function through the stabilization of protein folding. The pathway regulations of the last compounds seem unaffected by pressure. The ones of the glutamate biosynthesis pathway III (key enzyme: glutamate dehydrogenase, locus A7C91_RS06760) have upregulated expression at 0.1 MPa. This might indicate that piezolytes, and notably glutamate, are accumulated in cells at low hydrostatic pressures. The accumulation of glutamate in cells has been demonstrated in the piezophilic bacterium *Desulfovibrio hydrothermalis*, but in that case, the osmolyte accumulated under high hydrostatic pressures (Amrani et al., 2014). It has not been observed so far in *Thermococcales*. With regards to the compatible solutes that are known to accumulate in *Thermococcales* in response to pressure (mannosyl-glycerate (MG) and di-*myo*-1,3′-inositol phosphate (DIP)), the piezophilic strain *T. barophilus* produces more of the molecular chaperone MG under sub-optimal growth conditions (Cario et al., 2016). MG accumulation helps to increase protein rigidity at low pressure, but this compound is no longer necessary under pressure because pressure has a maintaining effect on proteins. *T. piezophilus,* however, does not possess the full MG synthesis pathway.

The overexpression of glutamate biosynthesis genes could suggest an overproduction of this metabolite at low pressures to help to support macromolecules conformation and functioning, an effect that could be physically counterbalanced by high pressures.

**Chemotaxis pathway and flagella**

A large majority of the genes coding for the chemotaxis pathway that sense the environment and relay it to the swimming organelle archaellum, are transcriptionally overexpressed at 50 MPa, and even more again at 90 MPa, compared to 0.1 MPa (Figure S1).

The overexpressed gene set of the chemotaxis system code for: (i) proteins involved in signal recognition and transduction such as (a) the methyl-accepting chemotaxis protein MCP (A7C91_RS01445), the central receptor of the signal and transducer, and (b) CheD (A7C91_RS01440), a glutamine deamidase playing a role in receptor maturation; (ii) proteins involved in excitation such as (a) the centerpiece histidine autokinase CheA (A7C91_RS01425 and A7C91_RS01435) which is the substrate of response regulators in its phosphohistidine form, and (b) its coupling protein CheW (A7C91_RS01400), (c) the methyltransferase CheR (A7C91_RS01410), an adaptational protein that alter the CheA activity to reset the system, and (d) the phosphatase CheC (A7C91_RS01430) involved in signal removal.

However, the chemotaxis signal transduction to the archaellum remains currently a mechanism poorly understood (Albers and Jarrell, 2015). The overexpressed genes of the archaellum (overexpression at 90 MPa and 50 MPa *vs* 0.1 MPa) code for three core components of the platform on which the archaellum assembly occurs, FlaJ (A7C91_RS01325), FlaI (A7C91_RS01330) and FlaH (A7C91_RS01335), and for the accessory protein FlaG (A7C91_RS01340).

Overexpression of chemotaxis genes (CheA, CheC, CheD and MCP) at a supra-optimal pressure for growth compared to an optimal pressure for growth has already been observed in the obligate piezophile *Pyrococcus yayanosii* (Michoud and Jebbar, 2016). In this other strain, an overexpression of chemotaxis genes was also observed at a sub-optimal pressure for growth.

The regulatory results observed for *T. piezophilus* indicate that chemotaxis and archaellum genes are hydrostatic-pressure responsive genes. We speculate that chemotaxis might allow the cell to migrate towards a niche with more favorable conditions when subjected to stressful high-pressure conditions. Pressure might have a direct effect on the engine that drives motility or on the genes regulating this process.

**S-Layer**

Genes coding for two protein subunits of the S-layer were overexpressed at sub-optimal pressure for growth (A7C91_RS04700 and A7C91_RS08075). This indicates that S-layer, which represents the outermost interaction zone with the natural environment, might play a role in cell stabilization as a function of hydrostatic pressure.

**CRISPR-Cas systems**

The CRISPR-Cas systems (clustered regularly interspaced short palindromic repeats and CRISPR associated *cas* genes) are also subjected to pressure regulations at the transcriptomic level, as already demonstrated in *P. yayanosii* (Michoud and Jebbar, 2016). Here, two clusters of genes corresponding to the CRISPR-*cas* type I-B system (A7C91_RS07605-25) and to the CRISPR-*cas* type III-A system (A7C91_RS00005-35) are overexpressed at 0.1 MPa in comparison to 50 and 90 MPa. Less noticeably, one gene encoding the Cas1b protein of the CRISPR-*cas* type I-B (A7C91_RS07585) and the genes encoding the CRISPR-associated protein Cas4 (A7C91_RS09870) and Cas2 (A7C91_RS04545) are overexpressed at 50 or at 90 MPa respectively. These results indicate that the CRISPR-Cas systems are sensitive to pressure variations. CRISPR-Cas systems are known roles to play role in DNA protection or in gene regulation (Aklujkar and Lovley, 2010;Bhaya et al., 2011). Gene regulation might be impacted by pressure in this strain.

**Stress response**

Even if a classical stress response was not observed in this model, very few genes (6 in total) involved in the stress response were still modulated by pressure (Supplementary Table 2). In brief, at 0.1 MPa, an overexpression of several enzymes associated with oxidative stress tolerance, such as rubrerythrin, rubredoxin and superoxide reductase (locus A7C91_RS10515-25) or peroxiredoxin (A7C91_RS10115) was observed. An overexpression of a gene coding for a chaperone protein of the thermosome (A7C91_RS05280) was detected at atmospheric pressure compared to 50 MPa. The thermosome might help stabilize proteins under stressful low-pressure conditions. Finally, it was also observed a decrease expression of the genes coding for the proteasome endopeptidases subunits alpha (A7C91_RS06075) and beta (A7C91_RS02745) at 0.1 MPa compared to 50MPa. This seems to be correlated to an increase expression of a proteasome assembly chaperone family protein (A7C91_RS03895) at this sub-optimal pressure.

**References**

Abe, F., and Horikoshi, K. (2000). Tryptophan permease gene TAT2 confers high-pressure growth in Saccharomyces cerevisiae. *Mol Cell Biol* 20**,** 8093-8102.

Aklujkar, M., and Lovley, D.R. (2010). Interference with histidyl-tRNA synthetase by a CRISPR spacer sequence as a factor in the evolution of Pelobacter carbinolicus. *BMC Evol Biol* 10**,** 230.

Albers, S.-V., and Jarrell, K.F. (2015). The archaellum: how Archaea swim. *Frontiers in Microbiology* 6.

Amrani, A., Bergon, A., Holota, H., Tamburini, C., Garel, M., Ollivier, B., Imbert, J., Dolla, A., and Pradel, N. (2014). Transcriptomics reveal several gene expression patterns in the piezophile Desulfovibrio hydrothermalis in response to hydrostatic pressure. *PLoS One* 9**,** e106831.

Bhaya, D., Davison, M., and Barrangou, R. (2011). CRISPR-Cas systems in bacteria and archaea: versatile small RNAs for adaptive defense and regulation. *Annu Rev Genet* 45**,** 273-297.

Cario, A., Jebbar, M., Thiel, A., Kervarec, N., and Oger, P.M. (2016). Molecular chaperone accumulation as a function of stress evidences adaptation to high hydrostatic pressure in the piezophilic archaeon Thermococcus barophilus. *Sci Rep* 6**,** 29483.

Empadinhas, N., and Da Costa, M.S. (2011). Diversity, biological roles and biosynthetic pathways for sugar-glycerate containing compatible solutes in bacteria and archaea. *Environmental Microbiology* 13**,** 2056-2077.

Gregory, G.J., and Boyd, E.F. (2021). Stressed out: Bacterial response to high salinity using compatible solute biosynthesis and uptake systems, lessons from Vibrionaceae. *Computational and Structural Biotechnology Journal* 19**,** 1014-1027.

Michoud, G., and Jebbar, M. (2016). High hydrostatic pressure adaptive strategies in an obligate piezophile Pyrococcus yayanosii. *Sci Rep* 6**,** 27289.

**Figure Legends**

**Figure S1: Network representation of differential gene expression between pressure conditions (represented by each color) and functional prediction for clusters showing gene overexpression**. Each node corresponds to the number of genes overexpressed in an experimental condition compared to another. The node size is proportional to the number of genes whose expression changes. The links represent the number of genes shared between nodes.

**Figure S2: Gene clusters regulated in hydrogenogenic/sulfidogenic metabolisms and their SurR binding motifs.** Each gene is colored according to its differential expression condition (0.1 MPa in blue, 50 MPa in green or 90 MPa in red). Triangles represent the SurR binding motifs. Red triangle is for the short binding motif (GTT*n*_3_AAC) and blue triangle is for the long one (GTT*n*_3_AAC*n*_5_GTT). The asterisk means that the motif carries a mutation.

**Figure S3. Hypothetical sensory pathway in *T. piezophilus* based on the genes found in the genome and known prokaryotic systems with analogous proteins (*Bacillus subtilis* for example).** All the proteins indicated on the diagram are encoded in the genome. Genes overexpressed at high pressures (RNA overexpression at 90 MPa and 50 MPa *vs* 0.1 MPa) are marked in red. Genes overexpressed at low pressures (RNA overexpression at 0.1 MPa *vs* 50 MPa and 90 MPa) are marked in green.
