# Supplementary figures and images for: The Piezo-Hyperthermophilic Archaeon Thermococcus piezophilus Regulates Its Energy Efficiency System to Cope With Large Hydrostatic Pressure Variations
Source: Front Microbiol. 2021 Nov 3;12:730231. doi: 10.3389/fmicb.2021.730231 (PMC8595942; doi:10.3389/fmicb.2021.730231)

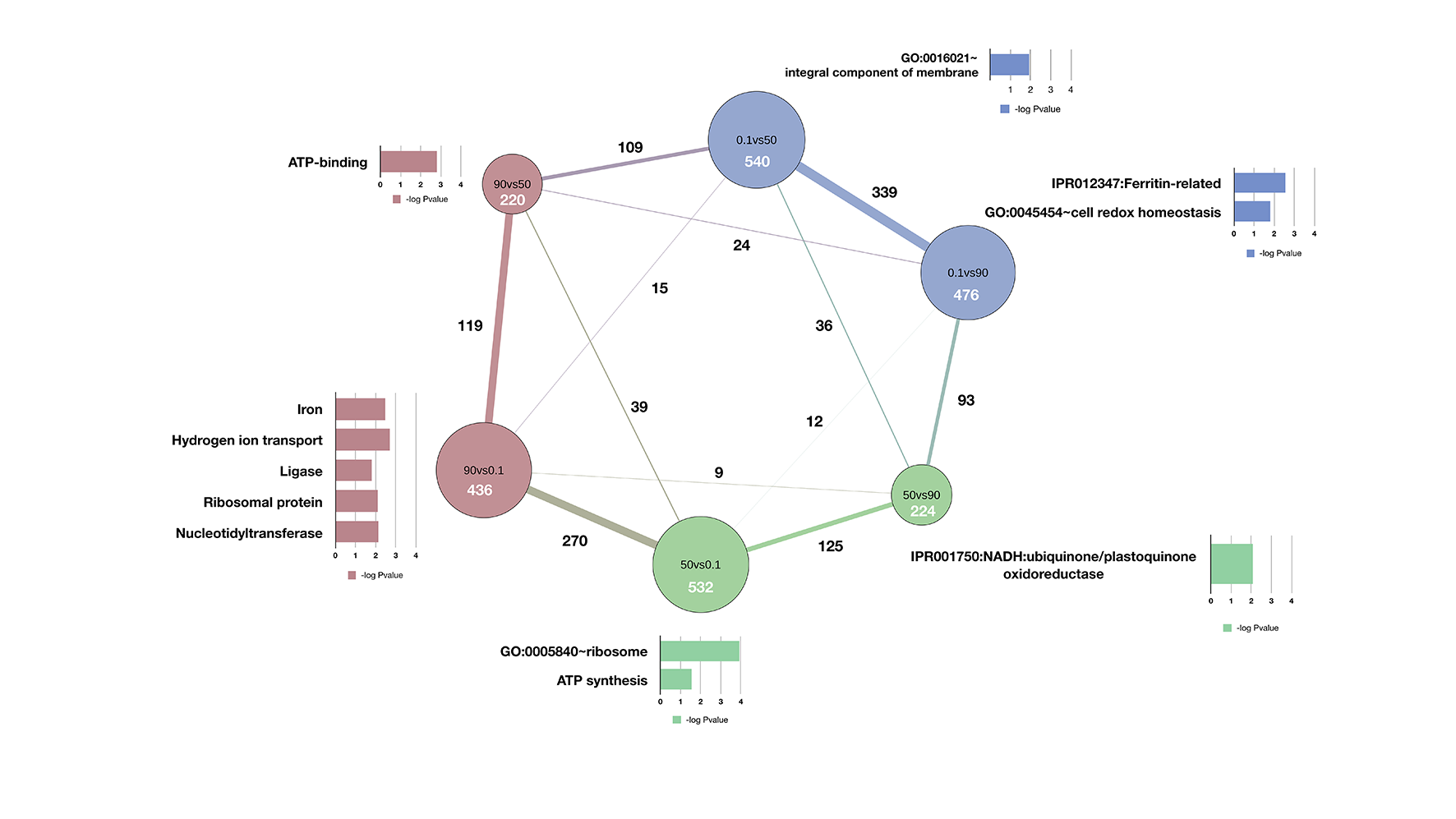

Supplement: Supplementary file 2 [file Image_1.TIF]

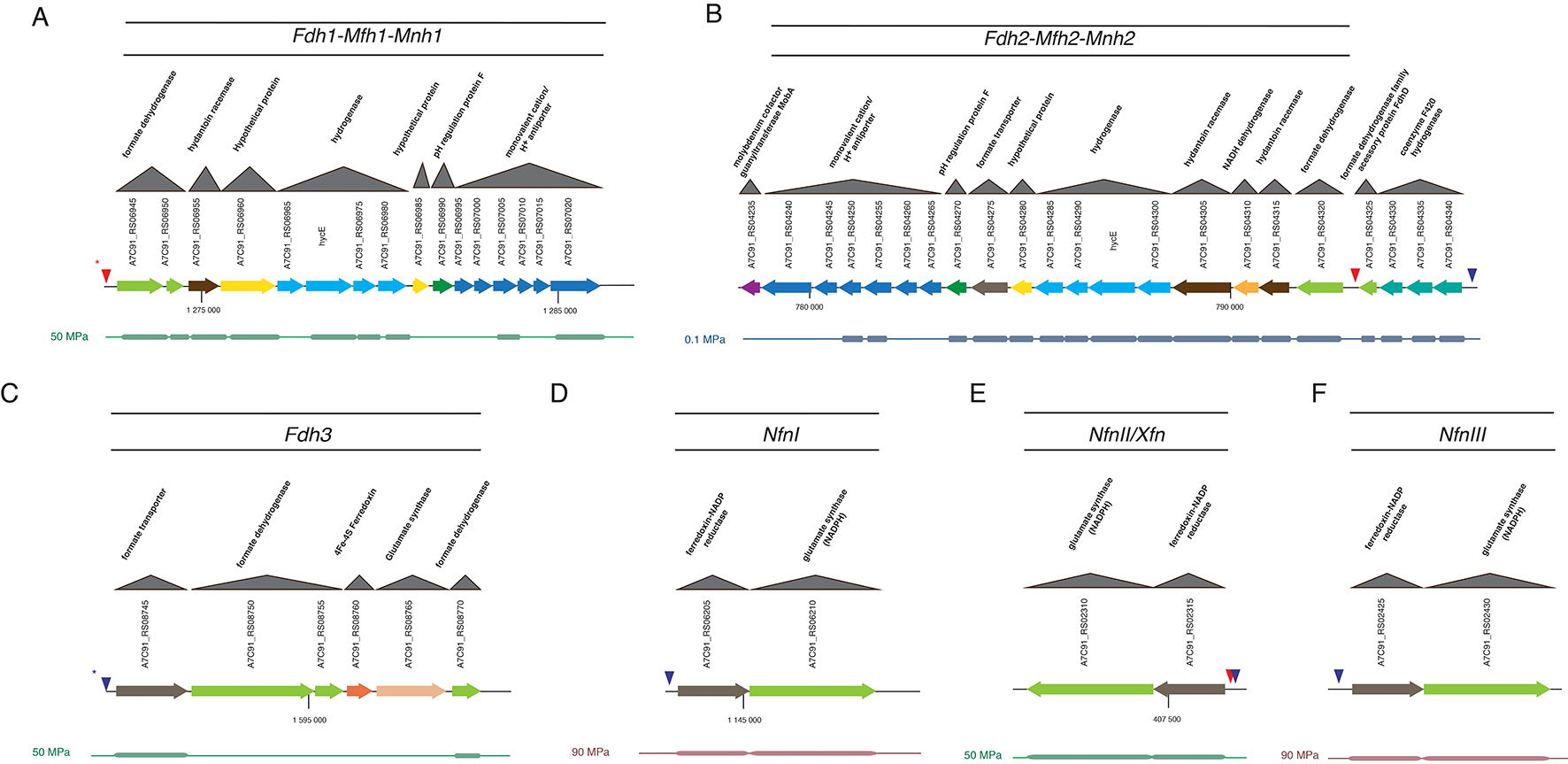

Supplement: Supplementary file 3 [file Image_2.TIF]

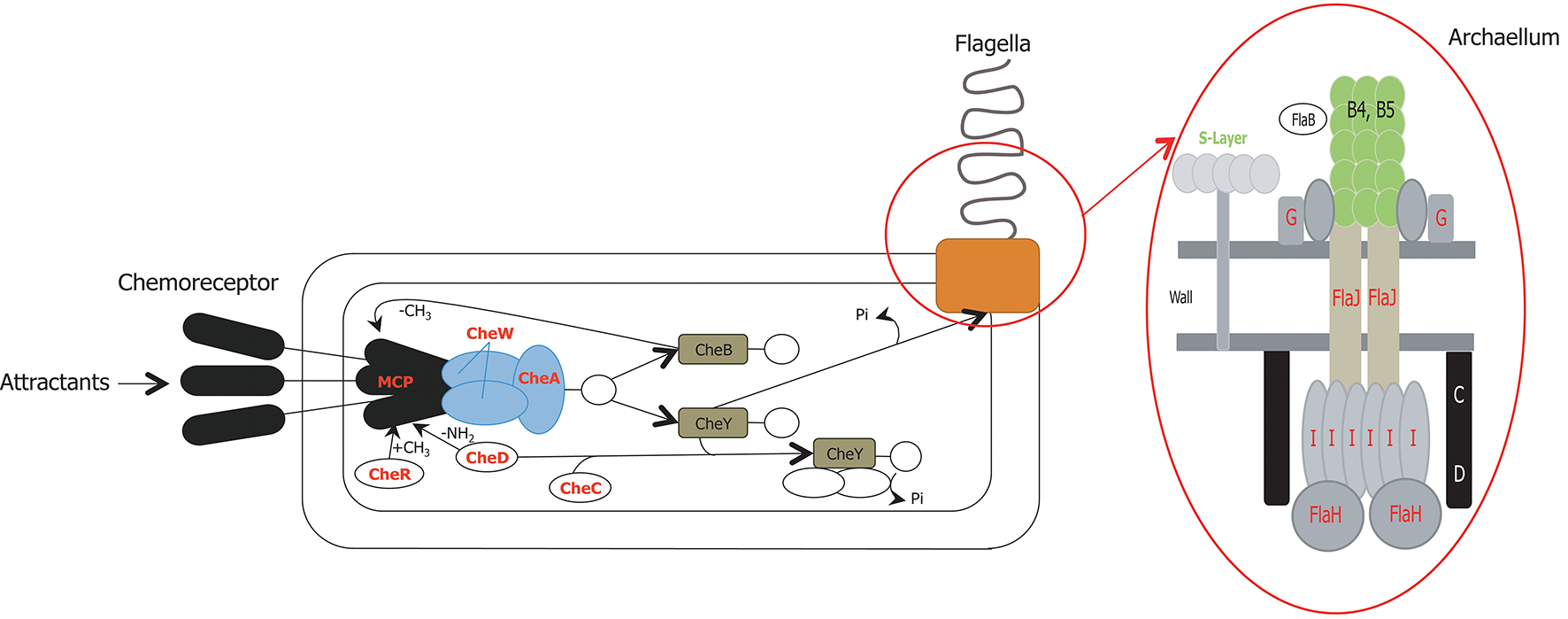

Supplement: Supplementary file 4 [file Image_3.TIF]
